# Healing Spaces: Designing Physical Environments to Optimize Health, Wellbeing, and Performance

**DOI:** 10.3390/ijerph17041155

**Published:** 2020-02-12

**Authors:** Altaf Engineer, Aletheia Ida, Esther M. Sternberg

**Affiliations:** 1 School of Architecture, University of Arizona, Tucson, AZ 85721, USA; aengineer@email.arizona.edu (A.E.); aida@email.arizona.edu (A.I.); 2 UArizona Institute on Place, Wellbeing & Performance, University of Arizona, Tucson, AZ 85711, USA; 3 Andrew Weil Center for Integrative Medicine, University of Arizona, Tucson, AZ 85711, USA

**Keywords:** human health, built environment, urban open space, forest healing, wellbeing, psychology, physiology

## Abstract

This Special Issue on Healing Spaces includes eight articles consisting of studies at the interface between design and health. The articles address some of the latest findings using state-of-the-art technologies, important outcomes for human health and wellbeing, and suggest exciting directions for the future of this research field.

The field of design and health, previously the purview of healthcare design professionals, has reached a new turning point where health impacts are becoming a focal point for designing environments on all scales. Many factors, including economic and societal, have contributed to this trend, but a large contributor is the proliferation of non-invasive wearable and stationary technologies measuring both health and environmental factors, which provide objective evidence for the real-time impacts of the built environment on many aspects of health, wellbeing, and performance. Research conducted with emerging tools is allowing for the discovery of human health variables in correlation to built environment conditions in expansive new ways. Each paper in this edited collection utilizes such technologies, knowledge from medical science, and sophisticated data analytics to discover relationships between environments and human wellbeing. 

This Special Issue of “Healing Spaces: Designing Physical Environments to Optimize Health, Wellbeing and Performance” in the *International Journal of Environmental Research and Public Health* (*IJERPH*) includes articles that address a spectrum of human health measures in different contexts. The techniques and methods vary, ranging from electroencephalography (EEG) devices to record frontal alpha symmetry (FAA) values, to correlational human subject surveys that assess mood states and other extrinsic and intrinsic human wellbeing factors. In some cases, the human subject research data is collected in real-time in the settings of interest, while in others the data is collected in a pre- and post-setting experience through laboratory testing. The settings and contexts tested also vary, ranging from urban public spaces to natural forests. 

The eight articles published in this issue focus on objective outcomes for human health and wellbeing based on measurements, which in turn leads to implications for the design of built environments for better health and wellbeing. This Special Issue provides both foundational knowledge for an emerging field of research as well as specialized results for design application. 

In the first paper, Olszewska-Guizzo et al. [1] discuss mental health outcomes from exposure to green spaces in urban areas. The authors test the accuracy of different methods for predicting positive mental health and wellbeing outcomes from urban landscape exposures. They also discuss the specific features of urban green spaces that may be most beneficial for mental health and wellbeing. The results of the study inform prevention and intervention measures for mental health, future research in the field, and design guidelines for optimal urban green spaces. 

Lyu et al. [2] study how bamboo forest therapy impacts immune system responses and psychophysiology of male college students. While bamboo forest therapy is identified as a fast-growing form of stress management, there is a knowledge gap in its specific health benefits, as the authors indicate. Some of the important findings of their study include an increase in positive mood states along with a reduction in negative mood states, and a decrease in heart rate, blood pressure, and corticosterone levels in the male participants exposed to forest environments. The authors, conclude that a three-day bamboo forest therapy session improves immune function, and physiological and psychological well-being in their participant cohort. Importantly, they recommend further studies to evaluate impacts on cardiovascular disease, hypertension, and cancer. 

Plans et al. [3] study the relationship between the density of green spaces and cardiovascular risk factors and whether this relationship is different for male and female residents in the city of Madrid, Spain. The cardiovascular risk factors studied include obesity, diabetes, hypertension, and high cholesterol. The findings reveal a moderate association between these risk factors, except for obesity, and the density of green spaces within different proximities (buffer sizes) for females, but not for males. More research on gender differences and their relationship to green spaces of different buffer sizes and cardiovascular health is therefore much-needed, as per the authors. The findings of this study, nonetheless, provide evidence for policy-makers wishing to create healthier environments in cities and reduce gender inequities.

Molina-García et al. [4] study the role of neighborhood characteristics in influencing physically active and sedentary behaviors in university students—a topic that has not been studied before, according to the authors. The authors find associations between neighborhood-built environments and socioeconomic status with active commuting, leisure-time physical activity, and sedentary behavior among university students. They discuss the implications of these findings, which include the design of university residential environments to promote walkability, available transportation, and exercise in college students. 

Chien et al. [5] discuss the benefits of urban open spaces on human health. Specifically, they examine the associations between the proximity to open spaces and adult renal function. The results reveal that a lower prevalence of chronic kidney disease is associated with proximity to open space among adults in Taiwan without hypertension or impaired fasting glucose. This paper highlights the positive association between open spaces and human physiology and complements the first paper, which shows the positive relationship between green spaces and mental health in urban areas. Additionally, the findings hold much significance for countries with a high population density, such as Taiwan, since it makes a stronger case for more open spaces to improve the health of residents. 

Takayama et al. [6] examine and compare the restorative effects of urban and forest settings on people. They find forest settings to have higher restorative properties than urban irrespective of individual traits, thereby highlighting the greater psychological and physiological benefits of forest environments. The authors call for more research on the relationship between forest settings and individual traits, and conclude by emphasizing the importance of developing forest experience programs suited for different individual trait types. The findings of this study highlight the effectiveness of forest therapy at combating daily stressors in urban life. As per the authors, this study could also be used to develop short-term forest staying programs for better psychological health in urban dwellers. 

Shepley et al. [7] conduct an in-depth literature review to reveal the relationship between the presence of urban green spaces and the frequency of violent crime. Using a qualitative method, they find that green interventions in built urban environments, such as vegetated streets, walkways, community gardens, or simply the amount of tree cover, resulted in a reduction in crime. While the results for the relationship between city parks and undeveloped green areas and crime were inconclusive, the authors recommend more meta-analyses and qualitative studies on the topic so that city governments and communities may use the data to support more effective interventions to mitigate violent crime in urban settings. 

Devos et al. [8], in the last paper of this Special Issue, emphasize the importance of acoustic environments to support persons with dementia. From a review of key concepts related to soundscapes, cognitive deficits, and other related behaviors, the authors propose a new framework for the composition and improvement of acoustic environments in dementia care environments. This framework consists of acoustic stimuli to influence moods, triggering feelings of safety, and other beneficial responses for residents with dementia. Optimal acoustic design for healthy spaces is often given less importance than visual or haptic design. This paper, therefore, makes an important contribution to the field.

Seven of the eight research studies focus on the urban and natural environments, using either open space or forest conditions as the spatial modality for human wellbeing impact. The eighth article is the only paper focusing on indoor memory care environments, though more specifically on the soundscape as an environment. Since papers were not targeted or solicited for this Special Issue in any particular domain, the preponderance of focus on urban and natural environments may reflect the well-established and long history of health studies at the urban planning scale, in contrast to the relatively recent advent of objective health measures at the individual building scale, beyond removing toxins from such environments. As the field of design and health continues to expand beyond healthcare facilities and public open space, we anticipate more human health research will emerge for varying indoor environments and programmatic uses. We envision that all spaces of the designed built environment, whether indoor, outdoor, urban, or natural, have the potential to contribute to human wellbeing and healing.

While each study reported here includes a relatively low number of participants, in combination, they provide increasing evidence for the health benefits of green spaces, whether urban or forest. The fact that these studies spanned the globe, from Spain, China, Taiwan, Singapore, to Japan and the US, is also indicative of the universal benefits of green spaces, regardless of culture or location. The articles in this special issue demonstrate how the built environment directly or indirectly affects human psychology, physiology, and overall wellbeing. We hope that this will stimulate more research in the burgeoning field of design and health. The interactions between humans and their environments are complex, and involve individual traits besides social, cultural, and behavioral issues. We are excited to see how more innovations in bio-sensing technology can help researchers address these issues to improve overall human health, wellbeing, and performance. As researchers continue to address knowledge gaps and take on new challenges, we look forward to mounting evidence for the health effects of built and natural environments at all scales and building types, impacting future directions in education, practice, and policy for the built environment. 
